# Commentary on: The Role of Nasal Fat Preservation in Upper Lid Surgery and Assessment With the FACE-Q Questionnaire: Innovations in Upper Blepharoplasty

**DOI:** 10.1093/asjof/ojae097

**Published:** 2024-11-04

**Authors:** Gabriele C Miotto

See the Original Article here.

It is a pleasure to be invited to comment on the article entitled “The Role of Nasal Fat Preservation in Upper Lid Surgery and Assessment With the FACE-Q Questionnaire: Innovations in Upper Blepharoplasty.”^1^ The authors describe their upper blepharoplasty technique utilizing nasal fat pad preservation in 11 patients over a period of 4 years using retrospective chart review. They are to be commended for highlighting the importance of volume preservation in upper eyelid surgery.

Over the last 2 decades, our understanding that volume loss is a major component of periorbital aging has become increasingly accepted.^2,3^ Modern eyelid surgery aims to preserve and enhance eyelid volume by various techniques, including muscle preservation, fat preservation, and the utilization of autologous fat grafting, not only in the upper eyelid but also in the whole periorbital area.^4,5^ The rejuvenated look our patients desire consists of periorbital beautification and volumetric reshaping, and no longer the standard removal of skin and fat.

It has been shown by Massry that nasal fat pad preservation in upper blepharoplasty plays a significant role in improving upper eyelid shape.^6^ Fat preservation helps maintain the natural volume and contour of the upper eyelid. The nasal fat pad is distinctively lighter in color, a fibrous type of fat, and found very medially in the upper eyelid. The medial aspect of the central fat pad can be confused with the nasal fat pad, as the later sits deeper behind the orbital septum and the central fat pad is more superficial and often larger, sitting in the preaponeurotic plane.

Releasing the nasal fat pad from its attachments is usually a critical step in transposing this fat pad. When done incompletely, it can cause tethering of the fat and incomplete gliding laterally. From the video presented in the author’s manuscript, it seems that the consistency, color, and easiness of gliding of the fat transposed are more compatible with the medial aspect of the central fat pad (preaponeurotic fat pad) than the nasal fat pad. Is it possible that the authors are transposing the medial aspect of the central fat pad, which is more superficial, through the preaponeurotic space? Or did the nasal fat migrated back to the nasal space since it was not fixed to any structures? In both postoperative images, there is basically unchanged volume and bulging of the nasal fat pad.

Upper eyelid ptosis can be a potential complication of repositioning of upper eyelid fat pad because of potential weight over the levator aponeurosis. Securing the transposed fat pedicle to the arcus marginalis periosteum, orbital septum, or undersurface of the orbicularis muscle has been an important component of the described transposition techniques to prevent undue weight over the levator aponeurosis. In this paper, the authors excluded patients with upper eyelid ptosis from their cohort of patients. However, it seems that patient in Figure 5 may have developed bilateral mechanical ptosis postoperatively after the procedure, as seen by the difference between before and after MRD1 and upper eyelid shape.

FACE-Q is a validated patient-reported outcome measure specifically designed to assess outcomes in facial aesthetic surgery and minimally invasive treatments. Its role in outcome metrics for facial surgery is multifaceted and significant. FACE-Q has been shown to be a reliable and valid tool for measuring patient-reported outcomes in different types of facial surgeries, such as rhinoplasty, blepharoplasty, and facial bone contouring, demonstrating significant improvements in patient satisfaction and quality of life following these procedures.^7-9^

The authors administered the FACE-Q Upper Lid questionnaire preoperatively and at the 6-month postoperative to assess patient satisfaction and results, which was positive in the present study. However, the authors refrained from conducting statistical evaluation of their data because of the small sample size. They also did not compare their results with FACE-Q results of patients undergoing blepharoplasty only. The effect of blepharoplasty alone is known to be positive on FACE-Q evaluation.^7-9^ Without an appropriate sample size or statistical analysis, the author's conclusions may not be accurate, especially in the absence of a comparison group.

In summary, the role of nasal fat pad preservation in upper blepharoplasty to maintain the natural volume and contour of the upper eyelid is an important feature of modern eyelid surgery. The author's approach using fat preservation is supported by recent trends in blepharoplasty that emphasize volume preservation and conservative tissue removal. However, larger samples, statistical analysis, and comparative groups between techniques and attention to aesthetic outcomes are important aspects we should consider to advance our specialty while innovating to deliver consistent periorbital beautification for our patients.
