# Correction to: Commentary on: The Role of Nasal Fat Preservation in Upper Lid Surgery and Assessment With the FACE-Q Questionnaire: Innovations in Upper Blepharoplasty

**DOI:** 10.1093/asjof/ojaf007

**Published:** 2025-02-04

This is a correction to: Gabriele C Miotto, Commentary on: The Role of Nasal Fat Preservation in Upper Lid Surgery and Assessment With the FACE-Q Questionnaire: Innovations in Upper Blepharoplasty, *Aesthetic Surgery Journal Open Forum*, Volume 6, 2024, https://doi.org/10.1093/asjof/ojae097

In the originally published article, the paper being commented on was missed as the first of the **REFERENCES** list. This is now added to that list in the article (“1. Nicodemi EM, Taraschi F. The Role of Nasal Fat Preservation in Upper Lid Surgery and Assessment With the Face-Q Questionnaire: Innovations in Upper Blepharoplasty. *Aesthet Surg J Open Forum*. 2024;6:ojae051. doi: 10.1093/asjof/ojae051”). Original reference “1” is emended to be “2”, and all other references respectively renumbered in the list and throughout the paper.

